# Pathological Technique

**Published:** 1898-12

**Authors:** 


**Pathological Technique.** By Frank Burr Mallory, M.D., and James Homer Wright, M.D. Pp. 397. London: The Rebman Publishing Co. (Ltd.). Philadelphia: W. B. Saunders. 1898.

We have here an excellent guide to the best methods of performing *post-mortem* examinations, inspecting gross lesions, and conducting bacteriological, histological, and chemical examinations. The growing attention which is now being paid to bacteriological methods of diagnosis is evidenced by chapters on "Bacteriological Examinations at Autopsies, "Clinical Bacteriology," and "Examination of the Blood." Although the work generally is most thorough, some points—such as the methods of best obtaining *post-mortem* cultures from the lung, or from vegetations in acute endocarditis—are treated very scantily. The technique, too, recommended for Widal's reaction is of somewhat a rough-and-ready nature.

The information on stains and staining methods is very full, and the recommendation of compressed carbon-dioxide instead of ether for freezing, the former being the cheaper and quicker method, is worthy of attention.

The chief value of the work to student and practitioner lies in the fact that the statement of the various methods employed in the solution of pathological problems is presented, not from one point of view only, but equally from the chemical, histological, bacteriological, and macroscopic standpoints.

